# Society's Misconceptions About Intra-articular Injections: A Cross-Sectional Study From Saudi Arabia

**DOI:** 10.7759/cureus.50285

**Published:** 2023-12-10

**Authors:** Abdullah Alshahrani, Saud M Alzahrani, Abdulmalik B Albaker, Ali H Alkhaldi, Ismail Almogbil, Naif M Alshaeri, Raghad Mahdi M Al-Awn, Mohammad A Alharbi

**Affiliations:** 1 Orthopedic Surgery, Medicine, Qassim University, Qassim, SAU; 2 Orthopaedic Surgery, Prince Meshari Bin Saud General Baljarshi Hospital, Al Baha, SAU; 3 Orthopaedics, Medicine, Majmaah University, Majmaah, SAU; 4 Medicine, South Al-Qunfudhah Hospital, Al Qunfudhah, SAU; 5 Surgery, Unaizah College of Medicine and Medical Sciencese, Qassim University, Buraidah, SAU; 6 Orthopedic Surgery, South Al-Qunfudhah Hospital, Al Qunfudhah, SAU; 7 Medicine and Surgery, King Khalid University Hospital, Abha, SAU; 8 Orthopaedic Surgery, Imam Mohammad Ibn Saud Islamic University, Riyadh, SAU

**Keywords:** society's misconceptions, local steroid injection, knee osteo-arthritis, intra-articular steroid injections, multiple intra-articular injections

## Abstract

Objective

This cross-sectional study aimed to identify and analyze misconceptions prevalent in Saudi Arabian society regarding intra-articular injections and their sources. It also sought to explore factors influencing patients' decision-making in the context of osteoarthritis (OA) treatment.

Methods

A sample of 405 adult participants from various educational backgrounds residing in Saudi Arabia completed a self-administered questionnaire. The survey focused on participants' knowledge about intra-articular injections, their sources of information, and their beliefs and perceptions related to these injections.

Results

The study unveiled a significant lack of awareness among the participants, with almost half (48.4%) reporting no knowledge of intra-articular injections. Of all, 16.1% held misconceptions about these injections, including believing that they are painful or they liquify the bone. Participants expressed varied thoughts on the effects of intra-articular injections, with 69.6% associating them with short-term pain relief. While 60.2% disagreed that intra-articular injections could provide long-term pain relief, 65.2% believed that these injections could delay the need for surgery. The source of knowledge varied, with 34.4% relying on orthopedics and 32.5% on friends and relatives. In those who had received intra-articular injections (n = 62), 83.9% cited the desire to delay or avoid surgery as a reason. While 56.5% were very satisfied with their doctor's guidance, 46.8% expressed concerns about relying on injections in the future. Furthermore, educational levels were correlated with knowledge, highlighting the need for more accessible and tailored patient education materials. However, even among those with a university education, misconceptions persisted. Effective patient-doctor communication was associated with secondary or university education (χ² = 11.05, p = 0.011).

Conclusion

The prevalence of misconceptions regarding intra-articular injections in Saudi Arabia underscores the need for comprehensive patient education and improved communication between healthcare providers and patients. Addressing these misconceptions is essential for empowering patients to make informed decisions about their healthcare and enhancing the overall quality of care they receive. This study's findings have implications not only for Saudi Arabia but also for healthcare systems globally, emphasizing the significance of patient-centered care and informed decision-making.

## Introduction

Osteoarthritis (OA), often referred to as degenerative joint disease, primary OA, wear-and-tear arthritis, or age-related arthritis, stands as a global health concern that significantly impacts the quality of life of affected individuals [1,2]. This multifaceted condition is particularly detrimental when it affects the knee joint, referred to as knee OA (KOA). KOA is characterized by persistent pain, muscle weakness, and a subsequent decline in physical function, leading to a diminished quality of life for those afflicted [3-5]. As one of the most prevalent types of arthritis, KOA is a widespread public health concern, necessitating a deeper understanding of its prevalence, risk factors, and the therapeutic modalities employed to manage it [1,6].

In Saudi Arabia, as in many parts of the world, the burden of OA has grown with the aging population [7]. Epidemiological data from Saudi Arabia have shown that the prevalence of OA increases with age, with a notable rise to 30.8% among individuals aged 46-55 and a striking 60.6% among those aged 66-75 [8]. Age remains a prominent endogenous risk factor for KOA, along with other factors such as sex, family history, ethnicity (with a higher prevalence in individuals of European heritage), and hormonal changes associated with menopause [9]. Exogenous risk factors, including macrotrauma, recurrent microtrauma, obesity, joint replacement surgery, and certain lifestyle choices, are additional contributors to the development of KOA [1,3,9].

The pathophysiology of KOA primarily involves the accumulation of mechanical stresses on the knee joint, leading to the breakdown of articular cartilage [1,10]. This degenerative process arises from a combination of increased degradation of the extracellular matrix (ECM), decreased production of ECM, and the death of chondrocytes. It is noteworthy that several members of the matrix metalloproteinase (MMP) and a disintegrin and metalloproteinase with thrombospondin motifs (ADAMTS) gene families have been implicated in the cartilage ECM degradation process [10].

In the early radiographic stages of KOA, non-invasive treatments are commonly recommended, including relative rest, oral anti-inflammatory and analgesic medications, and physical therapy [11]. However, when pain persists, intra-articular injections of various therapeutic agents, such as platelet-rich plasma (PRP), corticosteroids, and hyaluronic acid (HA), become necessary before considering surgical intervention. Although numerous pharmaceutical agents, including glucocorticoids and nonsteroidal anti-inflammatory drugs (NSAIDs), have been investigated for the management of KOA, none have provided a comprehensive cure and have often been associated with side effects [12]. Recent developments in the field of OA management include novel approaches such as anti-cytokine therapy, gene therapy, growth factor delivery, stem-cell therapy, and new lubricants, such as lubricin, offering promising avenues for exploration [12].

Intra-articular injections have emerged as a final conservative treatment option when pharmacological interventions prove ineffective [3]. Unlike systemic pharmacological treatments that can affect healthy tissues and organs and are primarily aimed at symptom management, certain newer intra-articular therapies target the underlying pathophysiological processes of KOA while improving symptoms [11]. This shift toward more targeted and effective interventions aligns with the concept of "molecular orthopedics," emphasizing the importance of addressing the fundamental causes of KOA, including cartilage loss and the associated biological processes [13].

The present study sets out to explore the landscape of KOA in Saudi Arabia, shedding light on its prevalence, risk factors, and perceptions of patients regarding intra-articular injections, a pivotal element in the management of KOA.

## Materials and methods

Study design

This cross-sectional survey was designed to investigate the prevalence of misconceptions in Saudi Arabian society concerning intra-articular injections and their sources, as well as to explore the factors influencing patients' decision-making regarding the use of intra-articular injections in the treatment of OA.

Study setting

The study was conducted in healthcare facilities in Saudi Arabia. Data were collected from participants attending orthopedic clinics at hospitals and primary healthcare centers, reflecting the real-world context where individuals seek medical advice and treatment for OA.

Target population and sampling

The target population for this study comprised all adult residents of Saudi Arabia, both male and female. A stratified sampling technique was employed to ensure the representation of various age groups, educational levels, and other demographic factors. The sample size was determined using a single population proportion formula, which yielded a minimum required sample size of 384 participants. To account for potential non-responses or incomplete data, a total of 405 participants were included in the study.

Data gathering instrument

Data were collected through the use of a self-administered, pre-tested questionnaire. The questionnaire was developed specifically for this study and was designed to comprehensively assess participants' knowledge, perceptions, and beliefs related to intra-articular injections. The questionnaire's validity and reliability were ensured through a comprehensive pre-testing process, involving a pilot study with a small sample size. The questionnaire was initially drafted in English, and then it underwent translation into Arabic. Back-translation was performed to verify the accuracy of the translation. It included sections that inquired about demographic information, knowledge of intra-articular injections, the sources of knowledge, and perceptions about the injections. Additionally, it examined factors influencing participants' decision-making processes, such as their level of trust in healthcare providers and satisfaction with the guidance received.

Inclusion and exclusion criteria

The inclusion criteria for participant selection in this study encompassed adults aged 18 years and above who actively sought orthopedic treatment specifically for knee pain, regardless of the cause (sports injury, trauma, mechanical, inflammatory, infective, metabolic, neoplastic).

Exclusion criteria comprised individuals aged below 18 years, students, physicians from other specialties, and those with cognitive impairments or communication difficulties. These criteria were established to ensure that the study's participants were relevant to the research objectives and that they could provide meaningful responses to the questionnaire that would reflect the society's status.

Data collection procedure

Data collection was carried out over a period of six months. Participants attending nine orthopedic clinics at the selected healthcare facilities were approached by trained data collectors who explained the study's objectives and procedures. Those who met the inclusion criteria and agreed to participate were provided with the self-administered questionnaire. Participants completed the questionnaire independently, ensuring privacy and confidentiality. Data collectors were available to clarify any questions or uncertainties that participants may have had.

Data analysis

The collected data were entered into Microsoft Excel 2016 for initial coding and organization. Subsequently, the dataset was analyzed using the Statistical Product and Service Solutions (SPSS) version 26 (IBM SPSS Statistics for Windows, Armonk, NY). Descriptive statistics were employed to summarize the data, including frequencies and percentages. Chi-square tests were used to examine associations and relationships between variables, such as educational level and knowledge of intra-articular injections. Statistical significance was set at a p-value of less than 0.05.

Ethical considerations

Ethical approval for the study was obtained from the HA-01-R-088 Majmaah University Institutional Review Board (approval number: MUREC-Jan. 29/COM-2023/5-6). All participants provided informed consent before participating, ensuring their voluntary and informed involvement in the research. Confidentiality and anonymity were assured, and the collected data were solely used for the purpose of this study.

## Results

Table 1 provides an insightful overview of the characteristics of the study participants and the sources of their knowledge regarding intra-articular injections. The data, collected from a total of 405 subjects, reveal a balanced distribution of gender, with 50.9% female and 49.1% male participants. The age distribution demonstrates a diverse group, with 29.6% falling within the 40-50 years category, 29.4% aged 50-70, 34.6% aged less than 40, and 6.4% aged over 70. The overwhelming majority of participants are of Saudi nationality, comprising 99% of the total sample, while non-Saudi participants constitute a mere 1%. Regarding marital status, the majority of participants are married (88.9%), followed by 6.7% who are widows and 4.4% who are divorced. In terms of educational level, the data reflect a varied range, with 18.5% classified as illiterate, 10.1% having primary or intermediate education, 22.2% having a secondary education, and 49.1% having a university degree or higher.

**Table 1:** Characters of participants and source of knowledge (n=405).

Parameter		Frequency (%)
Gender	Female	206 (50.9%)
	Male	199 (49.1%)
Age, y	40-50	120 (29.6%)
	50-70	119 (29.4%)
	Less than 40	140 (34.6%)
	More than 70	26 (6.4%)
Nationality	Saudi	401 (99%)
	Non-Saudi	4 (1%)
Marital status	Widow	27 (6.7%)
	Married	360 (88.9%)
	Divorced	18 (4.4%)
Educational level	Illiterate	75 (18.5%)
	Primary or intermediate education	41 (10.1%)
	Secondary education	90 (22.2%)
	University or more	199 (49.1%)
How long have you been following the orthopedic clinic for knee pain?	Never or less than 6 months	183 (45.2%)
	More than 6 months	91 (22.5%)
	More than 1 year	131 (32.3%)
Do you know about intra-articular injections?	No	196 (48.4%)
	Yes	209 (51.6%)
If yes, what is the source of knowledge? (n=209)	Friends and relatives	68 (32.5%)
	Orthopedician	72 (34.4%)
	Healthcare workers	8 (3.8%)
	TV, Google, articles, and social media	61 (29.2%)

Furthermore, the data reveal information about the participants' duration of following orthopedic clinics for knee pain. Notably, 45.2% reported never or less than six months of clinic attendance, while 22.5% had been attending for more than six months, and 32.3% for over a year. When it comes to awareness of intra-articular injections, 51.6% of participants reported being informed, while 48.4% were not. Among those who were aware, the primary sources of knowledge were friends and relatives (32.5%), orthopedics (34.4%), healthcare workers (3.8%), and various media sources such as TV, articles, and social media (29.2%).

Table 2 delves into the participants' knowledge regarding intra-articular injections and their attitudes toward this treatment modality. The data reveal that a considerable portion of the participants (57.5%) were not aware of any types of intra-articular injections, with 19.3% mentioning HA, 8.6% identifying HA and corticosteroids, and 14.6% recognizing corticosteroids. Furthermore, participants held varying beliefs about the nature of intra-articular injections, with 23% perceiving them as safe when used at proper intervals, 61% admitting a lack of knowledge, and 5.7% deeming them harmful with potential side effects. A significant majority (69.6%) believed that intra-articular injections provide short-term pain relief, 20.7% considered them ineffective, and 9.6% believed they had the same efficacy as surgical treatment.

**Table 2:** Knowledge of participants toward intra-articular injections (n=405).

Parameter		Frequency (%)
Types of intra-articular injections that you know	Hyaluronic acid	78 (19.3%)
	Hyaluronic acid and corticosteroids	35 (8.6%)
	Corticosteroids	59 (14.6%)
	None	233 (57.5%)
Thoughts on intra-articular injections	Scary injections that liquify the bone when used frequently	16 (4%)
	Injection inside the bone	26 (6.4%)
	Safe injections when used at proper intervals	93 (23%)
	Don't know	247 (61%)
	Harmful with side effects	23 (5.7%)
What is the effect of intra-articular injections?	Short-term pain relief	282 (69.6%)
	Not effective	84 (20.7%)
	Has the same efficacy as the surgical treatment	39 (9.6%)
If your orthopedician advised you to have intra-articular injections …	I will trust them and accept the treatment	231 (57%)
	I will ask for an alternative	174 (43%)
Do you know that cortisone medications are used to treat pain, asthma, inflammation, skin conditions, rheumatism, allergies, and cancers?	I partially know that	146 (36%)
	I know that well	117 (28.9%)
	Didn't know at all	142 (35.1%)
Do you know that the response to injections varies from one person to another?	Don't know	161 (39.8%)
	I know	244 (60.2%)
Intra-articular injections can provide long term pain relief	Agree	161 (39.8%)
	Disagree	244 (60.2%)
Do you believe that intra-articular injections can soften and damage bones and cause tendon and joint injuries?	Agree	141 (34.8%)
	Disagree	264 (65.2%)
Do you believe that intra-articular injections can delay the need for surgery	Agree	264 (65.2%)
	Disagree	141 (34.8%)
Do you agree with those who say, "The results of injections vary from one doctor to another"	Disagree	137 (33.8%)
	Agree	268 (66.2%)

Participants' trust in orthopedicians' advice regarding intra-articular injections was explored, with 57% expressing trust and willingness to accept the treatment, while 43% would seek an alternative. The data also investigated participants' knowledge of the varied uses of cortisone medications, with 36% having partial knowledge, 28.9% knowing them well, and 35.1% having no knowledge. Moreover, awareness of the variability in individual responses to injections was noted, with 60.2% of participants acknowledging this fact. In terms of beliefs about intra-articular injections, 39.8% agreed that they could provide long-term pain relief, while 60.2% disagreed. Furthermore, 34.8% agreed that intra-articular injections could cause harm to bones, tendons, and joints, while 65.2% disagreed. A majority (65.2%) believed that intra-articular injections could delay the need for surgery, whereas 34.8% disagreed. Participants were also asked if they agreed with the statement, "The results of injections vary from one doctor to another," with 66.2% agreeing and 33.8% disagreeing.

Table 3 investigates the association between educational level and participants' knowledge of intra-articular injections. The data reveal several statistically significant relationships. For example, there is a notable relationship between the duration of following the orthopedic clinic for knee pain and educational level (χ² = 26.847, p = 0.000). Participants who had less than six months of clinic attendance were more likely to have higher educational levels, while those with more than one year of attendance were more evenly distributed across educational levels. A significant association is also observed between knowledge of intra-articular injections and educational level (χ² = 16.204, p = 0.001). Participants with higher educational levels were more likely to be aware of intra-articular injections. A similar association exists between the source of knowledge and educational level (χ² = 30.628, p = 0.000). Those with higher educational levels were more likely to acquire knowledge from orthopedicians, while those with lower educational levels were more likely to rely on friends, relatives, or media sources.

**Table 3:** Educational level in association with knowledge of participants toward intra-articular injections (n=405).

Parameter		Educational level				X^2	P-value
		Illiterate	Primary or intermediate education	Secondary education	University or more		
How long have you been following the orthopedic clinic for knee pain?	Less than 6 months	14 (7.7%)	21 (11.5%)	45 (24.6%)	103 (56.3%)	26.847	0.000
	More than 6 months	26 (28.6%)	7 (7.7%)	20 (22%)	38 (41.8%)		
	More than 1 year	35 (26.7%)	13 (9.9%)	25 (19.1%)	58 (44.3%)		
Do you know about intra-articular injections?	No	49 (25%)	13 (6.6%)	48 (24.5%)	86 (43.9%)	16.204	0.001
	Yes	26 (12.4%)	28 (13.4%)	42 (20.1%)	113 (54.1%)		
If yes, what is the source of knowledge? (n=209)	Friends and relatives	11 (16.2%)	13 (19.1%)	7 (10.3%)	37 (54.4%)	30.628	0.000
	Orthopedician	14 (19.4%)	11 (15.3%)	14 (19.4%)	33 (45.8%)		
	Healthcare workers	0 (0%)	0 (0%)	0 (0%)	8 (100%)		
	TV, articles, and social media	1 (1.6%)	4 (6.6%)	21 (34.4%)	35 (57.4%)		
Types of intra-articular injections that you know	Hyaluronic acid	4 (5.1%)	10 (12.8%)	15 (19.2%)	49 (62.8%)	29.530	0.001
	Hyaluronic acid and corticosteroids	3 (8.6%)	4 (11.4%)	5 (14.3%)	23 (65.7%)		
	Corticosteroids	9 (15.3%)	2 (3.4%)	16 (27.1%)	32 (54.2%)		
	None	59 (25.3%)	25 (10.7%)	54 (23.2%)	95 (40.8%)		
Thoughts on intra-articular injections	Scary injections that liquify the bone when used frequently	1 (6.3%)	7 (43.8%)	0 (0%)	8 (50%)	69.572	0.000
	Injection inside the bone	3 (11.5%)	1 (3.8%)	0 (0%)	22 (84.6%)		
	Safe injections when used at proper intervals	10 (10.8%)	8 (8.6%)	24 (25.8%)	51 (54.8%)		
	Don't know	60 (24.3%)	24 (9.7%)	66 (26.7%)	97 (39.3%)		
	Harmful with side effects	1 (4.3%)	1 (4.3%)	0 (0%)	21 (91.3%)		
What is the effect of intra-articular injections?	Short-term pain relief	42 (14.9%)	26 (9.2%)	57 (20.2%)	157 (55.7%)	27.759	0.000
	Not effective	28 (33.3%)	12 (14.3%)	22 (26.2%)	22 (26.2%)		
	Has the same efficacy as the surgical treatment	5 (12.8%)	3 (7.7%)	11 (28.2%)	20 (51.3%)		
If your orthopedician advised you to have intra-articular injections …	I will trust them and accept the treatment	39 (16.9%)	17 (7.4%)	63 (27.3%)	112 (48.5%)	11.053	0.011
	I will ask for an alternative	36 (20.7%)	24 (13.8%)	27 (15.5%)	87 (50%)		
Do you know that cortisone medications are used to treat pain, asthma, inflammation, skin conditions, rheumatism, allergies, and cancers?	I partially know that	20 (13.7%)	9 (6.2%)	39 (26.7%)	78 (53.4%)	69.108	0.000
	I know that well	6 (5.1%)	12 (10.3%)	16 (13.7%)	83 (70.9%)		
	Didn't know at all	49 (34.5%)	20 (14.1%)	35 (24.6%)	38 (26.8%)		
Do you know that the response to injections varies from one person to another?	Don't know	55 (34.2%)	25 (15.5%)	31 (19.3%)	50 (31.1%)	61.860	0.000
	I know	20 (8.2%)	16 (6.6%)	59 (24.2%)	149 (61.1%)		
Intra-articular injections can provide long-term pain relief	Agree	20 (12.4%)	10 (6.2%)	48 (29.8%)	83 (51.6%)	16.651	0.001
	Disagree	55 (22.5%)	31 (12.7%)	42 (17.2%)	116 (47.5%)		
Do you believe that intra-articular injections can soften and damage bones and cause tendon and joint injuries?	Agree	25 (17.7%)	13 (9.2%)	20 (14.2%)	83 (58.9%)	10.703	0.001
	Disagree	50 (18.9%)	28 (10.6%)	70 (26.5%)	116 (43.9%)		
Do you believe that intra-articular injections can delay the need for surgery	Agree	33 (12.5%)	23 (8.7%)	52 (19.7%)	156 (59.1%)	33.759	0.000
	Disagree	42 (29.8%)	18 (12.8%)	38 (27%)	43 (30.5%)		
Do you agree with those who say, "The results of injections vary from one doctor to another"	Disagree	46 (33.6%)	19 (13.9%)	20 (14.6%)	52 (38%)	38.899	0.000
	Agree	29 (10.8%)	22 (8.2%)	70 (26.1%)	147 (54.9%)		
Have you ever received intra-articular injections as a treatment	No	65 (19%)	32 (9.3%)	78 (22.7%)	168 (49%)	1.903	0.593
	Yes	10 (16.1%)	9 (14.5%)	12 (19.4%)	31 (50%)		

The data further reveal that knowledge of types of intra-articular injections is significantly associated with educational level (χ² = 29.530, p = 0.001). Participants with a university degree or higher were more likely to be aware of the various types of injections. In terms of beliefs about intra-articular injections, there are significant associations with educational level in multiple aspects. Participants with higher educational levels were more likely to consider injections safe when used at proper intervals (χ² = 69.108, p = 0.000) were more aware of the varied response to injections (χ² = 61.860, p = 0.000) and were more likely to believe that intra-articular injections could provide long-term pain relief (χ² = 16.651, p = 0.001). Conversely, participants with lower educational levels were more likely to perceive injections as scary or harmful (χ² = 69.572, p = 0.000) and believe that injections could soften and damage bones and cause injuries (χ² = 10.703, p = 0.001). There is also a significant association between educational level and the belief that injections can delay the need for surgery (χ² = 33.759, p = 0.000), with participants having higher educational levels being more likely to hold this belief. Lastly, the data show that the agreement with the statement, "The results of injections vary from one doctor to another," is associated with educational level (χ² = 38.899, p = 0.000). Participants with higher educational levels were more likely to agree with this statement.

Table 4 offers a comprehensive look into the history of receiving intra-articular injections among the study participants. The data reveal that out of the total sample of 405 participants, 15.3% (62 individuals) reported having received intra-articular injections as a treatment. For those who had not received injections, the reasons for refusal were explored. Notably, 46.6% indicated that the needle size or injection pain was a factor in their decision to decline injections, while 53.4% stated that it was not a reason for their refusal. Additionally, when asked about their willingness to receive injections in the future if their pain increased, 39.1% expressed their willingness to consider it, 12.8% were opposed to the idea, and 48.1% remained unsure, highlighting the ambivalence of many participants toward future treatment with intra-articular injections.

**Table 4:** History of receiving intra-articular injections.

Parameter		Frequency (%)
Have you ever received intra-articular injections as a treatment (n=405)	No	343 (84.7%)
	Yes	62 (15.3%)
If no, is the needle size or injection pain a reason for your refusal to receive it (n=343)	No	183 (53.4%)
	Yes	160 (46.6%)
If no, are you willing to receive injections in the future if your pain increases (n=343)	No	44 (12.8%)
	Don't know	165 (48.1%)
	Yes	134 (39.1%)

In Table 5, which focuses on participants who had received intra-articular injections, the data delve into the reasons for undergoing this treatment. Among those who had received injections, the majority (59.7%) reported age-related cartilage wear (primary OA) as the reason, while 40.3% cited early cartilage wear for any reason (secondary OA). In cases of early cartilage wear, the leading causes were rheumatic diseases (52%), followed by sports injuries (20%), fractures (20%), and limb deviation or deformity (8%). Participants also revealed the frequency of injections they had received, with 41.9% having received injections once, 33.9% twice, 12.9% three times, and 11.3% more than three times. The location of injections was also documented, with 64.5% receiving injections on one side and 35.5% on both sides.

**Table 5:** History of participants who received intra-articular injections (n=62).

Parameter		Frequency (%)
What is the reason for taking the injection (n=62)	Early cartilage wear for any reason (secondary osteoarthritis)	25 (40.3%)
	Age-related cartilage wear (primary osteoarthritis)	37 (59.7%)
What is the reason for early cartilage wear (n=25)	Rheumatic diseases	13 (52%)
	Sports injury	5 (20%)
	Deviation and deformity in limbs	2 (8%)
	Fracture	5 (20%)
How many times have you received intra-articular injections (n=62)	More than three times	7 (11.3%)
	Three times	8 (12.9%)
	Once	26 (41.9%)
	Twice	21 (33.9%)
Did you receive the injection on one side or both sides (n=62)	Both sides	22 (35.5%)
	One side	40 (64.5%)
Below is a list of treatment methods using injections used in clinics. Please select the injection you received (n=62)	Both	11 (17.7%)
	Corticosteroids	20 (32.3%)
	Hyaluronic acid	31 (50%)
If you have been treated with intra-articular injections before, how often does your doctor explain the drug plan, treatment, and side effects (n=62)	Rarely explains them	10 (16.1%)
	Always explains them	29 (46.8%)
	Usually explains them	15 (24.2%)
	Only explains when I ask	8 (12.9%)
How satisfied are you with your doctor's guidance regarding the best treatment for you (n=62)	Very satisfied	35 (56.5%)
	Not satisfied	9 (14.5%)
	Neutral	18 (29%)
Was your desire to delay or avoid the operation the reason for receiving the injection (n=62)	No	10 (16.1%)
	Yes	52 (83.9%)
Do you have concerns about relying on injections in the future (n=62)	No	27 (43.5%)
	Yes	35 (56.5%)
If someone told you, "You will get used to it and its effectiveness will decrease over time," would you agree and reduce its use (n=62)	Disagree	19 (30.6%)
	Agree	43 (69.4%)
Do you agree with the statement, "Once you start intra-articular injections, you must take them forever” (n=62)	Disagree	33 (53.2%)
	Agree	29 (46.8%)
What is your expectation for a successful injection (n=62)	Rapid pain relief	35 (56.5%)
	Complete pain relief	13 (21%)
	Long term pain relief	14 (22.6%)
Do you think that your weight will increase with intra-articular injections (n=62)	I don't think so	37 (59.7%)
	I believe so	25 (40.3%)
Do you agree with those who say, "Cortisone injections in the joint do not change blood glucose levels" (n=62)	Disagree	29 (46.8%)
	Agree	33 (53.2%)
Do you agree with those who say that oily injections are safer and more effective than cortisone injections (n=62)	Disagree	27 (43.5%)
	Agree	35 (56.5%)

Furthermore, the data examined the specific types of injections received by these participants. Among the respondents, 50% had received hyaluronic acid injections, 32.3% received corticosteroid injections, and 17.7% had received both types. The participants' experiences with communication and guidance from their doctors were also explored. Approximately 46.8% reported that their doctors always explained the drug plan, treatment, and side effects; 24.2% stated that their doctors usually explained them; and 12.9% said their doctors only explained when asked. The majority of participants (56.5%) expressed being very satisfied with their doctor's guidance regarding the best treatment for them, 14.5% were not satisfied, and 29% were neutral.

The reasons for receiving injections were further probed, with a significant 83.9% indicating that their desire to delay or avoid surgery was the motivation behind receiving the injection. Concerns about relying on injections in the future were expressed by 56.5% of participants, while 43.5% had no such concerns. The data also investigated how participants would react if they were told that the effectiveness of injections would decrease over time. A substantial 69.4% agreed that they would reduce its use, while 30.6% disagreed. The belief that starting intra-articular injections necessitates taking them forever divided the participants, with 46.8% agreeing and 53.2% disagreeing.

Participants were also asked about their expectations regarding the effectiveness of injections, with 56.5% anticipating rapid pain relief, 21% expecting complete pain relief, and 22.6% hoping for long-term pain relief. Concerns about weight gain as a side effect of intra-articular injections were expressed by 40.3% of participants, while 59.7% did not believe it would cause weight gain. Moreover, participants' opinions regarding the impact of cortisone injections on blood glucose levels were divided, with 53.2% agreeing that it does not change blood glucose levels and 46.8% disagreeing. Lastly, participants were asked to share their views on whether oily injections are safer and more effective than cortisone injections, with 56.5% agreeing and 43.5% disagreeing.

## Discussion

This study aimed to shed light on the prevalent misconceptions within Saudi Arabian society concerning intra-articular injections, explore their sources, and discern the factors influencing patients' decision-making.

The survey reveals a substantial level of misconceptions among the study's participants. Nearly half of the respondents (48.4%) reported that they had no knowledge of intra-articular injections. This initial finding accentuates a knowledge deficit that may impact patients' decision-making regarding this medical intervention. In Saudi Arabia, where OA is a common ailment, a lack of awareness about intra-articular injections is concerning [14].

The prevalence of misconceptions about the types of intra-articular injections is also notable. For instance, a segment of the respondents believed that the injections liquify the bone. Such perceptions can significantly deter individuals from considering these treatments as a viable option, especially when the need arises [14,15].

Intra-articular injections, particularly for OA, are globally recognized as a valuable therapeutic approach, offering pain relief and potentially delaying the need for more invasive procedures [16]. However, misconceptions about their efficacy and safety are not unique to Saudi Arabia. Studies from various countries have reported similar misconceptions and knowledge gaps among patients [14,17,18].

Comparable studies in Saudi Arabia revealed that patients often hold incorrect beliefs about the composition and effects of intra-articular injections [14,17]. Such misconceptions, as in our study, had the potential to influence their decision-making regarding these injections. While the specific misconceptions may vary between countries, the underlying issue of inadequate patient education and the need for improved communication between healthcare providers and patients are universal concerns [18].

The association between educational levels and knowledge about intra-articular injections corroborates findings from previous studies [14,15,18]. Our study demonstrates that respondents with a university-level education were more likely to be aware of these injections. While higher education is generally linked to better health literacy, it is noteworthy that, even within this group, misconceptions still persist perhaps attributable to the source of knowledge. This finding highlights the need for more accessible and tailored patient education materials, regardless of educational background [15].

Akin to our results, other studies have reported a correlation between higher education and a better understanding of medical interventions. For example, a study found that higher education levels were linked to better knowledge about treatment options for joint pain. However, this should not lead to complacency; healthcare providers must ensure that patients from all educational backgrounds have access to comprehensive and comprehensible information [17].

Effective communication between healthcare providers and patients plays a pivotal role in dispelling misconceptions and fostering informed decision-making [2,7]. Our study indicates that many respondents were satisfied with their doctor's guidance. This is encouraging and suggests that doctors in Saudi Arabia are already taking steps to ensure that their patients are well-informed about the treatment.

Patients who reported higher satisfaction with their healthcare providers' communication were more likely to have accurate knowledge about medical treatments. Our study's findings align with this, suggesting that doctors can be effective agents in educating patients and dispelling misconceptions [15,17,18].

Study implications

The prevalence of misconceptions regarding intra-articular injections in Saudi Arabia poses significant challenges to patient-centered healthcare. Addressing these misconceptions requires a multifaceted approach. Healthcare providers must take on the role of educators, offering patients accurate and accessible information about these injections. Tailored interventions, including educational campaigns and materials, should be developed to suit patients from various educational backgrounds.

Furthermore, our findings highlight the importance of patient-doctor relationships. When patients have trust and confidence in their healthcare providers, they are more likely to rely on their guidance and make informed decisions. Enhancing the quality of patient-doctor communication is, therefore, a critical step toward addressing misconceptions.

Limitations

Despite its strengths, the study has certain limitations. The reliance on self-reported data is a potential limitation, as it may be subject to recall bias or social desirability bias. This could affect the accuracy of participants' responses and the overall reliability of the data. The study primarily identifies misconceptions but does not delve into the reasons behind them or assess their impact on patient behavior or healthcare outcomes. Finally, the study could benefit from further investigation into the sources of information that contribute to misconceptions about intra-articular injections.

## Conclusions

In conclusion, this study offers insights into the prevalence of misconceptions and their impact on patients' decision-making regarding intra-articular injections in Saudi Arabia. It highlights the need for patient education, the significance of healthcare providers in correcting misconceptions, and the role of communication in building patient trust. By addressing misconceptions, we can empower patients to make informed decisions about their healthcare and improve the overall quality of care they receive. This study contributes to the global understanding of misconceptions about medical treatments and their effects on healthcare decision-making, and its findings have relevance not only in Saudi Arabia but also in healthcare systems worldwide.

## Appendices

Questionnaire on the patient's perception of pain injection therapy

1. Your (patient) age?

A. Less than 40 years old

B. 40-50 years old

C. 50-70 years old

D. More than 70 years old

2. What is your gender?

A. Male

B. Female

3. How many years have you been attending the orthopedic clinic due to knee pain?

A. Less than 6 months

B. More than 6 months

C. Over 1 year but less than 3 years

D. More than 3 years

4. Have you ever received an injection as treatment?

A. Yes

B. No

If yes,

5. How many times you received an intraarticular injection as treatment

A. One

B. Two

C. Three

6. The intraarticular injection was received unilateral or bilateral

A. Unilateral

B. Bilateral

7. If you have been treated with injections before, is your physician good at describing its pharmacological, therapeutic, and side effects?

A. Always explained

B. Usually explained

C. Explained only when the patient asked

D. Did not explain

8. Have you heard about intraarticular injection?

A. Yes

B. No

9. If you have heard of intraarterial injections, who told you about them?

A. Mass media such as newspapers, news, and the Internet

B. Anesthesiologist

C. Physician in other specialties

D. Oriental doctor

E. Pharmacist

F. Friends or relatives

10. What do you think of intraarticular injection?

A. Scary injections that dissolve bones if used frequently

B. systematic complications

C. Safe injection without side effects if used at an appropriate time interval

D. I do not know

11. Which type of injection

A. Hyaluronic acid > go to question number

B. Steroid injection > go to question number

C. Both

12. Do you know that steroids are drugs that are used for pain treatment, as well as asthma, atopy, dermatitis, rheumatism, allergies and cancers?

A. I know it well

B. I know roughly

C. I do not know at all

13. If your doctor uses steroids for your treatment, are you fine with that?

A. I believe in the physician and agree with his treatment decision

B. I would ask for another treatment or ask to stop the steroids

14. Did you know that the response to injections varies from one patient to another before they are given?

A. Yes

B. No

15. Was your desire to delay or avoid the surgery the reason for receiving the injection?

A. Yes

B. No

16. Will you refuse to have the injection because of the size of the needle, or the pain involved?

A. Yes

B. No

17. Did you have a concern about becoming reliant on injections?

A. Yes

B. No

18. What did you expect from a successful injection?

A. Quick pain relief

B. Total pain relief

C. Lasting pain relief

19. What do you think about intra-articular injections?

A. It has the same effect as surgery

B. It’s a short-term relief for knee pain

C. Not useful

20. Do you think that long-term pain relief from intra-articular injections is possible?

A. Yes, I do

B. No, I don’t

21. Do you think that “intra-articular injections can help you to delay your surgery”?

A. I agree

B. I don’t believe that

22. Do you agree that intra-articular injections are only to relieve pain?

A. Yes

B. No

23. Do you think that “intra-articular injections soften (destroy) bones and deteriorate tendons and joints”?

A. I agree

B. I think it’s a myth

24. If someone came to you and says, “You will get used to it and it will become less effective,” would you agree and avoid using it?

A. Yes

B. No, I don’t believe it’s true

25. Do you think that you will gain weight with intra-articular injection?

A. Yes, I think so

B. No, I don’t think it will affect my weight

26. Do you agree with this statement “Once you start intraarticular injections, you’ll have to take them forever”?

A. I do

B. No, I don’t

27. Do you agree with this statement: “Intra-articular steroid injections do not result in systemic blood glucose changes"?

A. Yes, I do

B. No, I think it will affect the blood glucose level

28. Do you agree that “natural injections like hyaluronic acid or platelets rich plasma are much effective and safer than synthetic corticosteroids”?

A. Yes

B. No

29. Do you agree that “intra-articular injections only mask deeper problems by temporarily relieving symptoms”?

A. Yes

B. No

30. Do you agree that “the more expensive the injection, the more effective it is"?

A. Yes

B. No

31. Do you agree that “the injection results vary from doctor to doctor”?

A. Yes

B. No

32. How satisfied are you with the doctor’s guidance regarding the type of treatment that is best for you?

A. Completely satisfied

B. Neutral

C. Unsatisfied

33. When deciding whether an injection is the appropriate treatment, what sources do you trust to consult?

A. Physicians

B. The Internet

C. Experiences of those who received injections

D. Friends and family
